# The Safety and Efficacy of Daprodustat for Recipients in Peritransplant Period: a Single-Center Retrospective Study on Post-Transplant Anemia Management in Kidney Transplantation

**DOI:** 10.3389/ti.2025.15237

**Published:** 2025-08-21

**Authors:** Yu Sato, Hiroshi Noguchi, Shinsuke Kubo, Yu Hisadome, Keizo Kaku, Soichiro Tajima, Yasuhiro Okabe, Masafumi Nakamura

**Affiliations:** ^**1**^ Department of Surgery and Oncology, Graduate School of Medical Sciences, Kyushu University, Fukuoka, Japan; ^2^ Department of Pharmacy, Kyushu University Hospital, Fukuoka, Japan

**Keywords:** HIF-PH inhibitor, erythropoiesis-stimulating agent, renal transplantation, anemia, chronic kidney disease (CKD)

Dear Editors,

Post-transplant anemia (PTA) is a common complication after kidney transplantation, with a prevalence ranging from 38.6% to 45.6% [1]. PTA is associated with poor graft function and graft survival [1, 2]. While erythropoiesis-stimulating agents (ESAs) are widely used for PTA [3], hypoxia-inducible factor prolyl hydroxylase (HIF-PH) inhibitors have recently emerged as alternative oral therapies [4, 5]. Daprodustat is a once-daily oral HIF-PH inhibitor that stabilizes HIF-α subunits, including HIF-1α and HIF-2α, maintains their activity, and activates the downstream genes of HIF signaling [6]. HIF-2 induces renal and hepatic EPO synthesis in response to daprodustat, which stimulates erythropoiesis. Although HIF-PH inhibitors, including daprodustat, are widely used for anemia of chronic kidney disease, evidence for their use in the immediate post-transplant period remains limited.

We conducted a single-center retrospective study to assess the safety and efficacy of daprodustat, a HIF-PH inhibitor, in managing PTA during the peritransplant period. We included adult recipients of living-donor kidney transplants from June 2019 to March 2023 who received treatment for PTA within 1 week after transplantation. Patients who received daprodustat (n = 59) or ESAs (n = 74) were identified, and 42 patients from each group were matched using propensity scores calculated using preemptive transplantation, ABO incompatibility, and prior ESA use. The target hemoglobin was 10–12 g/dL. Both groups received iron supplementation as needed.

Hemoglobin levels declined initially in both groups, reaching a nadir in the first postoperative week, and subsequently increased over 12 weeks, with no significant difference between the groups (Figure 1A). There were also no significant differences in progression of renal function within 12 weeks after transplantation (Figure 1B). As hemoglobin increased, more than half of patients in the daprodustat group discontinued treatment within 12 weeks (Figure 1C). Three patients in the ESA group transitioned to HIF-PH inhibitors after 12 weeks. At 1 year, both hemoglobin levels and eGFR were comparable between groups; hemoglobin was 12.5 ± 1.6 g/dL in the daprodustat group versus 13.0 ± 1.7 g/dL in the ESA group (p = 0.205), and eGFR was 49.3 ± 15.5 mL/min/1.73 m^2^ versus 50.9 ± 14.0 mL/min/1.73 m^2^, respectively (p = 0.620).

**FIGURE 1:**
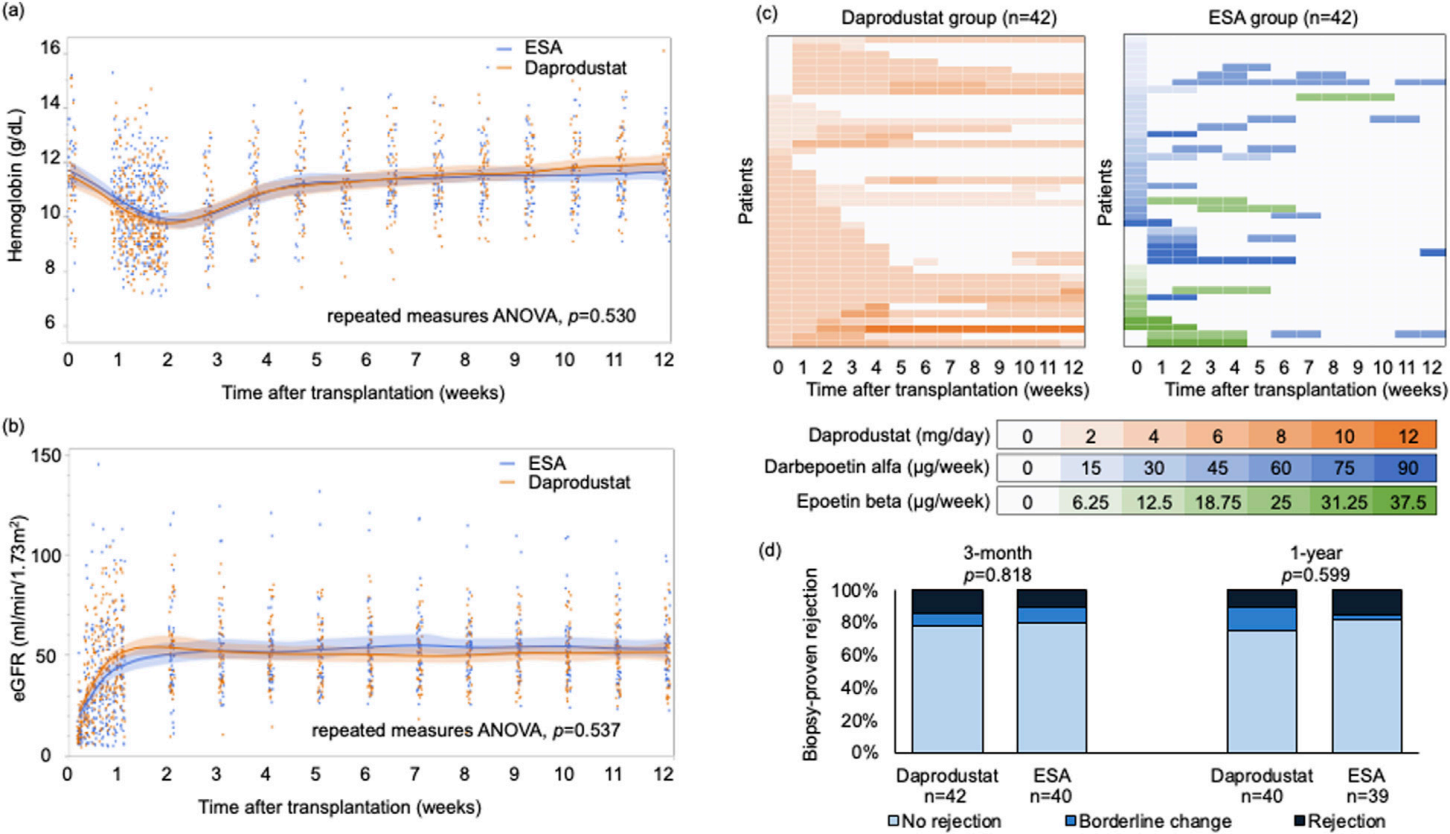
**(A)** Hemoglobin levels and **(B)** eGFR of the groups within 12 weeks after transplantation. Data are presented as mean values with 95% confidence intervals. Repeated measures analysis of variance showed no significant differences between the groups in either hemoglobin levels or eGFR. **(C)** Dose of daprodustat or ESAs of each patient. Vertical axis represents each patient, and color intensities indicate the dosage levels at each time point. Darker colors correspond to higher doses of daprodustat or ESAs. **(D)** Biopsy-proven rejection rates were compared between the groups. The biopsy results were classified in the order of no rejection, borderline change, and rejection, and compared with two-sided Mann-Whitney U test. ANOVA, analysis of variance; eGFR, estimated glomerular filtration rate; ESA, erythropoietin-stimulating agent.

Safety outcomes were also similar. One thromboembolic event occurred in the daprodustat group (pulmonary embolism), and one major adverse cardiovascular event (MACE) was observed in each group. No deaths or graft losses occurred. Rates of biopsy-proven rejection at 3 months and 1 year did not differ significantly (Figure 1D).

In our study, the effectiveness of daprodustat in maintaining hemoglobin and renal function was similar to ESAs during the early post-transplant period, suggesting that daprodustat can be safely initiated in kidney transplant recipients during peritransplant period. Because it is suggested that rapid hemoglobin elevation and iron deficiency induced by HIF-PH inhibitors may increase the risk of thrombotic events, monitoring hemoglobin and serum iron levels could be important to avoid hemoglobin overshoot and iron deficiency. In our institution, HIF-PH inhibitors were initiated during hospitalization, and patients underwent weekly monitoring for the first 3 months after transplantation. This allowed fine adjustment of HIF-PH inhibitor dosing to avoid hemoglobin overshoot. In addition, especially when using HIF-PH inhibitors, serum iron and ferritin are monitored, and iron supplementation is performed if needed. However, because a meta-analysis has shown that long-term use of HIF-PH inhibitors increases the risk of thrombotic events compared with ESAs [7], we should pay attention to patients requiring long-term use of daprodustat.

It is known that PTA is associated with impaired allograft function and increased mortality following kidney transplantation [1, 8]. Moreover, correcting PTA with ESAs has been shown to improve allograft function and prolong survival in prospective interventional trials [9, 10]. Hypoxia in the tubulointerstitium due to anemia may contribute to kidney allograft damage and the development of CKD in transplant recipients [9]. Therefore, early correction of PTA after transplantation may be crucial for the long-term prognosis of kidney allografts. Our study demonstrated that daprodustat treats PTA as effectively as ESAs, suggesting that correcting PTA with daprodustat could also improve kidney allograft outcomes similar to ESAs.

This study has several limitations. First, as this was a retrospective study, it was susceptible to selection bias. There were significant differences in background characteristics between the groups; therefore, we conducted a propensity score matching analysis to create comparable cohorts. Additionally, the retrospective design resulted in some missing data, including iron metabolism markers such as ferritin. Monitoring of iron markers is crucial for assessing and managing anemia; therefore, further investigation is needed after accumulating sufficient data. Second, we could only evaluate short-term outcomes within 1 year after transplantation. Given that nearly half of the patients in the daprodustat group discontinued daprodustat and a certain number of patients in the ESA group started taking daprodustat or other HIF-PH inhibitors even beyond 3 months after transplantation, we could not accurately assess the long-term effects of daprodustat on transplant outcomes. Despite these limitations, this study provides valuable insights into the safety and efficacy of perioperative daprodustat administration in kidney transplant recipients.

In conclusion, daprodustat can be safely used to manage anemia in kidney transplant recipients in the early post-transplantation period.

## Data Availability

The raw data supporting the conclusions of this article will be made available by the authors, without undue reservation.

## Abbreviations

eGFR, Estimated Glomerular Filtration Rate; ESA, Erythropoiesis-Stimulating Agent; HIF-PH, Hydroxylation-Inducible Factor Prolyl Hydroxylase; MACE, Major adverse cardiovascular event; PTA, Post-Transplant Anemia.
